# Emerging Trends and Translational Challenges in Drug and Vaccine Delivery †

**DOI:** 10.3390/pharmaceutics16010098

**Published:** 2024-01-11

**Authors:** Prashant Kumar, Vibhuti Agrahari

**Affiliations:** 1 Department of Pharmaceutical Chemistry, Vaccine Analytics and Formulation Center, University of Kansas, 2030 Becker Dr, Lawrence, KS 66047, USA; 2 Department of Pharmaceutical Sciences, University of Oklahoma Health Sciences Center, 1110 N. Stonewall Avenue, Oklahoma City, OK 73117, USA

Drug and vaccine delivery have received considerable attention in recent years. Many rationally designed innovative approaches are being explored to address the challenges related to safety, efficacy, patient compliance, and cost-effective means for existing and new therapeutics. The extensive assessment of drug delivery involves pre-formulation and physicochemical characterization, mechanistic biochemical pathways at the molecular level, pharmacological and toxicological evaluations, and detailed preclinical investigations. Recent advancements have evolved to address the limitations that emerged with the evolution of novel therapeutic modalities from simple small molecules to more complex macromolecules, including nucleic acids, peptides, proteins, antibodies, and conjugates [1]. There’s immense interest in exploring the in vitro and in vivo behavior of drugs and vaccines to overcome biological barriers to reach target sites, and in expeditious translation from the lab to a manufacturing scale [2]. This Special Issue on “Emerging Trends and Translational Challenges in Drug and Vaccine Delivery” is the collection of those efforts by several researchers to address the unmet need of advanced drug and vaccine delivery systems. The studies published in this Special Issue are summarized below and are valuable for the readers of Pharmaceutics and the scientific community working in the field of drug and vaccine delivery.

The first paper in this collection by Alkholief et al. demonstrated the use of dexamethasone-sodium-phosphate (DEX)-chitosan nanoparticles (CSNPs) coated with hyaluronic acid (HA) as a controlled release ocular delivery vehicle for the treatment of endotoxin-induced-uveitis (EIU) in a rabbit model [3]. The CSNPs were stable at 25 °C for 3 months and in vitro studies showed a similar DEX release in a range of 74–77% for uncoated and HA-coated nanoparticles. Drug-loaded CSNPs were safe for ocular applications and showed a noTable 10-fold increase in transcorneal flux and permeability of DEX in the case of HA-CSNPs vs. DEX-aqueous solution (DEX-AqS). The findings suggest improved delivery properties and promising anti-inflammatory effects of DEX-CSNPs in EIU rabbits with ocular bioavailability, with the half-life and ocular MRT0-inf of DEX being significantly higher than DEX-AqS.

Another study focused on extracellular nanovesicles (EVs) that have great potential as drug delivery systems for precision therapy but are limited due to technical challenges to purify and characterize the EVs. To address this issue, Nguyen et al. developed a 3D inner filter-based technique for the simple extraction of apoplastic fluid from blueberries, enabling EV purification [4]. The high drug loading capability and properties to modulate the release of proinflammatory cytokine IL-8 and total glutathione have enabled blueberry-derived EVs (BENVs) to be a promising edible multifunctional nano-bio-platform for future immunomodulatory therapies.

Vaccination is the most effective way to prevent infectious diseases but suffers from fading immunity requiring frequent boosters to maintain the immune response. In a novel approach, Kooji et al. demonstrated the effectiveness of a single injection with sustained-release microspheres as an alternative to the conventional multiple injection (prime-boost) immunization schedule of bovine serum albumin in terms of eliciting the same levels of IgG antibody response in mice [5]. The microspheres were designed based on two novel biodegradable multi-block copolymers with an opportunity to tailor the release profile in a range of 4 to 9 weeks by varying the polymer ratios.

Adjuvants are ingredients used in many vaccines to elicit a stronger immune response. In a recent study, Liang et al. demonstrated the use of formulated phospholipid 1,2-dimyristoyl-sn-glycero-3-phosphocholine (DMPC), a component of oil-in-water vaccine adjuvant emulsion (known as a stable emulsion or SE), as non-canonical agonists for murine and human TLR4 [6]. The effects of DMPC on human cells were proven but were less pronounced than the composition of emulsion oil and were dependent on the saturation, size, and headgroup of the phospholipid.

The next article is focused on drug loaded-microneedles, which are minimally invasive systems capable of painless delivery and offer dose-sparing benefits with a potential to replace hypodermal needles and oral routes of delivery. In this study, Faizi et al. developed a deferasirox-nanosuspension (DFS-NS) loaded with dissolving microneedles (DMN) for intradermal delivery for effective treatment of iron overload [7]. DFS-NSs were formulated by the wet media milling procedure using PVA and showed a 3-fold higher dissolution rate vs. pure DFS. The skin deposition studies showed significantly higher drug deposition from DFS-NSs loaded with polymeric dissolving microneedles (NS-DMN) as compared to DFS-NS transdermal patches without needles (DFS-NS-TP) or pure DFS-DMNs. Hence, the authors showed that loading DFS-NSs into novel DMN devices can be effectively used for transdermal delivery of sparingly soluble drugs, i.e., DFS in aqueous systems.

In another study, Peng et al. demonstrated the development of amphotericin B (AMB)- and levofloxacin (LVX)-loaded chitosan films for potential use in antimicrobial wound dressings [8]. An HPLC method developed by the authors measured 100% and 60% release of LVX and AMB, respectively, from the chitosan film after a week. An ex vivo deposition study showed that 20.96 ± 13.54 and 0.35 ± 0.04 of LVX and AMB, respectively, were deposited in porcine skin 24 h after application. Further, the films were able to inhibit the growth of *Candida albicans* and *Staphylococcus aureus*, demonstrating their antimicrobial applications.

Wang et al. in their recent review discussed the translational challenges and prospective solutions for implementing biomimetic delivery systems (BDSs) for therapeutic delivery [9]. BDSs are based on complex designs of biological structures and have emerged as a powerful tool for drug and vaccine delivery. This review provides recent advances in the development of BDSs, discusses the challenges faced in the translation of BDs from research to clinical applications, and presents emerging solutions, emphasized by real-world case studies.

Luo et al. provide insights into the development of organs-on-chips (OCs) and their impact on precision medicine and advanced system simulation [10]. OCs are devices with micro-physiological systems containing small tissues grown inside microfluidic chips with controlled cell microenvironments to study the pathophysiology and effect of drugs on the human body. OCs represent a faster, economical, and precise approach to study drug safety, efficacy, disease modelling and treatments with a potential to complement/replace traditional preclinical cell cultures, animal studies, and even human clinical trials.

Ingle and Fang in their recent review present an overview of the stability and delivery challenges of commercial nucleic acid (NA)-based therapeutics, including DNA, RNA, oligonucleotides, siRNA, miRNA, mRNA, small activating RNA, and gene therapies [11]. The review highlights NA-based therapeutics approved by the European Medicines Agency (EMA) and US Food and Drug Administration (US FDA) with a focus on the current progress in improving the stability, delivery, cost, and regulatory acceptance of these therapeutics.

There is significant interest in developing approaches to overcome the blood–brain barrier (BBB) for treatment of central nervous system (CNS) diseases. Meyer et al. in their recent review described novel developments to enable the treatment of CNS diseases with targeted drug delivery [12]. The review focuses on unfolding the full potential of novel therapeutic entities, i.e., gene therapy and degradomers, using innovative delivery systems for possible application in the treatment of CNS diseases.

In conclusion, this Special Issue converses through the translation of therapeutic delivery from discovery to large-scale production for pharmaceutical and biotechnology applications. The discussed strategies, including the use of polymeric nanoparticles, extracellular nanovesicles, sustained release microspheres, microneedles, polymeric biofilms, biomimetic delivery systems, new adjuvants, and organ-on-chips, possess great potential in addressing the limitations of drug and vaccine delivery. The editors express their gratitude for the interest and cooperation of the contributors and believe this Special Issue of Pharmaceutics would be an interesting addition to the scientific community engaged in drug delivery research.

## References

[1] Vargason A.M., Anselmo A.C., Mitragotri S. (2021). The Evolution of Commercial Drug Delivery Technologies. Nat. Biomed. Eng..

[2] Agrahari V., Kumar P. (2022). Novel Approaches for Overcoming Biological Barriers. Pharmaceutics.

[3] Alkholief M., Kalam M.A., Raish M., Ansari M.A., Alsaleh N.B., Almomen A., Ali R., Alshamsan A. (2023). Topical Sustained-Release Dexamethasone-Loaded Chitosan Nanoparticles: Assessment of Drug Delivery Efficiency in a Rabbit Model of Endotoxin-Induced Uveitis. Pharmaceutics.

[4] Nguyen T.N., Pham C.V., Chowdhury R., Patel S., Jaysawal S.K., Hou Y., Xu H., Jia L., Duan A., Tran P.H. (2023). Development of Blueberry-Derived Extracellular Nanovesicles for Immunomodulatory Therapy. Pharmaceutics.

[5] van der Kooij R.S., Beukema M., Huckriede A.L.W., Zuidema J., Steendam R., Frijlink H.W., Hinrichs W.L.J. (2023). A Single Injection with Sustained-Release Microspheres and a Prime-Boost Injection of Bovine Serum Albumin Elicit the Same Igg Antibody Response in Mice. Pharmaceutics.

[6] Liang H., Lykins W.R., Seydoux E., Guderian J.A., Phan T., Fox C.B., Orr M.T. (2022). Formulated Phospholipids as Non-Canonical Tlr4 Agonists. Pharmaceutics.

[7] Faizi H.S., Vora L.K., Nasiri M.I., Wu Y., Mishra D., Anjani Q.K., Paredes A.J., Thakur R.R.S., Minhas M.U., Donnelly R.F. (2022). Deferasirox Nanosuspension Loaded Dissolving Microneedles for Intradermal Delivery. Pharmaceutics.

[8] Peng K., Li M., Himawan A., Dominguez-Robles J., Vora L.K., Duncan R., Dai X., Zhang C., Zhao L., Li L. (2022). Amphotericin B- and Levofloxacin-Loaded Chitosan Films for Potential Use in Antimicrobial Wound Dressings: Analytical Method Development and Its Application. Pharmaceutics.

[9] Wang Z., Wang X., Xu W., Li Y., Lai R., Qiu X., Chen X., Chen Z., Mi B., Wu M. (2023). Translational Challenges and Prospective Solutions in the Implementation of Biomimetic Delivery Systems. Pharmaceutics.

[10] Luo Y., Li X., Zhao Y., Zhong W., Xing M., Lyu G. (2023). Development of Organs-on-Chips and Their Impact on Precision Medicine and Advanced System Simulation. Pharmaceutics.

[11] Ingle R.G., Fang W.J. (2023). An Overview of the Stability and Delivery Challenges of Commercial Nucleic Acid Therapeutics. Pharmaceutics.

[12] Meyer A.H., Feldsien T.M., Mezler M., Untucht C., Venugopalan R., Lefebvre D.R. (2023). Novel Developments to Enable Treatment of Cns Diseases with Targeted Drug Delivery. Pharmaceutics.

